# Unconditional cash transfer to reduce the burden of unmet surgical needs

**DOI:** 10.1016/j.amsu.2022.104185

**Published:** 2022-07-14

**Authors:** Robert B. Laverty, Rahul M. Jindal

**Affiliations:** Brooke Army Medical Center, San Antonio, TX, USA; Department of Surgery, Uniformed Services University, Indian Institute of Public Health, Gandhinagar, Fulbright-Nehru Distinguished Chair, India

**Keywords:** Unconditional cash transfer, Unmet surgical needs, India, Low- and middle-income countries

## Abstract

Recent years have seen scandals involving international humanitarian organizations. Short term surgical missions from high to low- and middle-income countries have been criticized as ‘parachute’ missions. There are significant surgical unmet needs in low- and middle-income countries. Universal health coverage has been underutilized in low- and middle-income countries for surgical conditions. We suggest a two-fold solution: first, restructuring of aid organizations by splitting them into smaller units to make them transparent and responsive to local needs. Secondly, unconditional cash transfer directly to beneficiaries giving them a choice to select physician and hospital for surgical treatment.

## 1 Introduction

Prestigious and well-funded international humanitarian organizations have been embroiled in scandal over recent years. One investigation revealed that OXFAM [1] aid workers held sex parties with prostitutes in Haiti. Additionally, the organization allegedly failed to warn other charities of the misconduct after the workers had transferred. British newspapers reported that key executives in Save the Children, a venerable organization, had sexually harassed female employees [2]. Over 1,000 current and former staff of Médecins Sans Frontières (MSF) signed an open letter alleging that this organization was institutionally racist, and reinforced colonialism and white supremacy in its humanitarian work. The statement accused MSF of failing to acknowledge racism, poor hiring practices, toxic workplace culture and “dehumanizing” programs, run by a “privileged white minority” workforce [3]. The Washington Post ran an expose on the U.S. government's botched attempts to curtail corruption in Afghanistan which was managed by State Department and USAID; misappropriated funds totaled $934 - $978 billion [4]. Internal issues abound at USAID as well; staffers alleged that the leadership was not sympathetic after the death of George Floyd in police custody and the subsequent racial reckoning. Approximately 2,400 USAID employees signed on to a letter to the USAID administrator urging him to address racial equality and discrimination.

Short term surgical missions and electives from organizations in high-income countries (HIC) who travel to low-middle-income countries (LMIC) have been criticized as ‘parachute’ missions and a form of colonization with no or minimal reciprocity to trainees in LMIC [5,6]. We suggest a two-fold solution. First, these aid organizations should undergo a radical restructuring by splitting into smaller units to make them more transparent and responsive to local needs. Secondly, unconditional cash transfer should be initiated to beneficiaries bypassing entrenched bureaucracy after appropriate community consultation [6].

## 2 Surgical unmet needs in India

The estimated unmet surgical needs are high in low-middle-income countries (LMIC), including India. In our community-based cross-sectional survey of over 2,000 low-income households in Ahmedabad, India, nearly 16% of households reported getting medical advice for surgical procedures over the previous year, of which almost 37% had an unmet need [7]. The findings suggested that financial constraints, fear of surgery, and higher opportunity costs for the patient and/or caretakers while undergoing surgical procedures were the leading reasons for not availing of surgical treatment. Most of these households received various government social security schemes qualifying them for government health scheme benefits that offer free healthcare.

## 3 Conceptualization of SATHI – an intermediary community health worker

In order to reduce the incidence of unmet surgical needs, we conceptualized '*SATHI*’ (Surgical Accredited & Trained Healthcare Initiative) as an intermediary channel through which those in need could be linked to service providers. These personnel will help patients attain the benefits of health schemes and offer counseling through trust-building and elimination of fear of surgical procedures. They would provide emergency first aid for minor injuries commonly seen in daily wage laborers, identifying common surgical diseases, timely referral to local hospitals, and ensure compliance with post-surgical follow-up. The concept of intermediary community workers similar to ASHA [8] (https://en.wikipedia.org/wiki/Accredited_Social_Health_Activist) and SEVAK (www.sevakproject.org) [9], has a proven track record in several medical conditions in India. The *SATHI* worker will guide patients to hospitals who participate in Universal Health Coverage (UHC) such as Rashtriya Swasthya Bima Yojana (https://www.india.gov.in/spotlight/rashtriya-swasthya-bima-yojana). For those patients who prefer to go to private surgeons of their choice, evidence suggests that the financial expenditure under the schemes are inadequate. Furthermore, a large proportion of the needy are not aware about these schemes to begin with [10].

## 4 Unconditional cash transfer to reduce unmet surgical needs

Unconditional cash transfer has been effective in several countries affected by humanitarian catastrophes but not utilized for alleviating planned, non-emergent surgical procedures in LMIC. Stein et al. [11] used both quantitative and qualitative data to understand the impact of unconditional cash transfer to refugees in the setting of the COVID-19 pandemic and subsequent cuts to monthly aid. Through telephonic surveys in 1,200 households, the authors investigated the effect of these cash transfers on COVID-19 specific preventative health practices, food security and psychological well-being. The cash transfers were not found to significantly impact preventative measures against COVID-19, however, households receiving the cash transfer were more food secure, had better psychological well-being and were more likely to seek healthcare in the private health facilities as compared with control households. Doocy et al. [12] examined the impact of cash and voucher assistance on the prevention of child acute malnutrition during the Somalian food crisis of 2017–2018. Households were given $450 USD in food vouchers or a mix of in-kind food, vouchers and cash. Investigators then measured changes in diet and acute malnutrition for children aged 6–59 months from these households. There were no significant changes in dietary diversity, meal frequency, or the proportion of children with minimum acceptable diet for either intervention group. Another study examined three different assistance programs: in-kind food commodities, food vouchers, and unrestricted vouchers in Syria [13]. They found that both in-kind food assistance and voucher programs showed positive effects on food security, however, no intervention was successful in improving all outcomes measured. Voucher programs were found to be more cost-efficient than in-kind food assistance, and more cost-effective for increasing household food consumption.

Doocy and Tappis [14] in a systematic review examined the effectiveness, efficiency and implementation of cash transfers in humanitarian settings. They summarized evidence from 5 studies of effects, 10 studies of efficiency and 108 studies of barriers and facilitators to implementation of cash-based humanitarian assistance. They found that unconditional cash transfers and vouchers improved household food security in conflict-affected populations and maintained food security in drought-affected populations.

## 5 Conclusions

We suggest a radical overall in the structure of aid organizations by splitting them into smaller units to make them more transparent and responsive to local needs. An even innovative solution would be unconditional cash transfer directly to the people in need of surgical attention in LMIC bypassing entrenched bureaucracy and giving the option to beneficiaries the choice of their surgeon and hospital. There are some examples that both conditional and unconditional cash transfers can make positive impacts on the lives of the poor [15]. However, agencies must factor in beneficiary selection methods for implementation of cash-based interventions. Large-scale studies are still needed to demonstrate the amount, method and metrics of success for this specific intervention.

## Provenance and peer review

Not commissioned, externally peer reviewed.

## Disclaimer

The opinions or assertions contained herein are the private ones of the author/speaker and are not to be construed as official or reflecting the views of the Department of Defense, the Uniformed Services University of the Health Sciences or any other agency of the U.S. Government. No financial conflict of interest exists for any of the authors.

## Sources of funding for your research

None.

## Ethical approval

Not indicated.

## Consent

Not applicable.

## Author contribution

All authors listed have made a substantial, direct, and intellectual contribution to the work and approved it for publication.

## Registration of research studies

1. Name of the registry:
2. Unique Identifying number or registration ID:
3. Hyperlink to your specific registration (must be publicly accessible and will be checked):

## Guarantor

Dr Jindal.

## Declaration of competing interest

None.
